# The Surface Age of Sputnik Planum, Pluto, Must Be Less than 10 Million Years

**DOI:** 10.1371/journal.pone.0147386

**Published:** 2016-01-20

**Authors:** David E. Trilling

**Affiliations:** 1 Department of Physics and Astronomy, Northern Arizona University, Flagstaff, AZ, United States of America; 2 Lowell Observatory, Flagstaff, AZ, United States of America; SETI Institute, UNITED STATES

## Abstract

Data from the New Horizons mission to Pluto show no craters on Sputnik Planum down to the detection limit (2 km for low resolution data, 625 m for high resolution data). The number of small Kuiper Belt Objects that should be impacting Pluto is known to some degree from various astronomical surveys. We combine these geological and telescopic observations to make an order of magnitude estimate that the surface age of Sputnik Planum must be less than 10 million years. This maximum surface age is surprisingly young and implies that this area of Pluto must be undergoing active resurfacing, presumably through some cryo-geophysical process. We discuss three possible resurfacing mechanisms and the implications of each one for Pluto’s physical properties.

## Introduction

Recent images of Pluto from the New Horizons spacecraft [1] have revealed a number of surprises. Chief among these is the complete lack of detectable craters in the region informally known as Sputnik Planum (SP). (See for example http://tinyurl.com/ph8bcr5 and http://tinyurl.com/qfto99p for low and high resolution images, respectively.) Several workers [2–4] made predictions of the expected crater distribution of Pluto, but none of these predicted the absence of craters observed for SP. We can make an order of magnitude estimate of the age of this region of Pluto’s surface from our knowledge of the population of small Kuiper Belt Objects (KBOs) in which Pluto orbits.

## Methods

A number of observatories on the ground and in space have been used to measure the size distribution of KBOs, which is usually expressed as *N*(<*H*), the number of KBOs larger than *H* (typically measured per square degree). (*H* is the Solar System absolute magnitude, which is the magnitude an object would have 1 AU from the Earth and 1 AU from the Sun at zero phase; small values of *H* correspond to big objects. Diameter estimates derived from *H* are only approximate because the albedos of the KBOs are not known.) Two recent results [5, 6] find *N*(<*H*) of 10^1.7^ for *H* ≤ 12 (around 11 km diameter). At *H* ≤ 17 (around 1 km), the estimate is 10^4^ KBOs per square degree [7]. These results can be extrapolated to estimate that there are 10^5.8^ KBOs larger than *H* = 22 (100 meters) per square degree.

The ecliptic plane is something like 10 degrees in height, so the total area of the ecliptic is 360 degrees times 10 degrees, or 3600 deg^2^.

Pluto sweeps out a torus around the Sun as it orbits; the volume of this torus is *C*_*Pl*_ *πr*^2^, where *C*_*Pl*_ is the circumference of Pluto’s orbit (to first order, 40 AU) and *r* is Pluto’s radius. Gravitational focusing for Pluto is negligible. The volume occupied by the Kuiper Belt is *C*_*KB*_ *πR*^2^, where *C*_*KB*_ is approximately 40 AU, and *R* is the width of the main part of the Kuiper Belt, around 2 AU. Thus, the fraction of the Kuiper Belt that Pluto sweeps out is simply (*r*/*R*)^2^, which is around 1.5 × 10^−11^. This fraction must be reduced by the ratio of the mean impact velocity (estimated to be around 2 km/sec by [2]) to Pluto’s orbital velocity of 4.7 km/sec to account for the fact that Pluto is not orbiting in a static field of impacting KBOs, but rather that the surrounding KBO swarm is also orbiting the Sun.

The number of impacts onto Pluto per Pluto year as a function of size (that is, *H*), on average, is therefore given by

$$N\left(<H\right)\;{\text{deg}}^{-2}\times 3600\;{\text{deg}}^{2}\times \frac{2.0\;\text{km}/\text{sec}}{4.7\;\text{km}/\text{sec}}\times 1.5\times 10^{-11}.$$ (1)

One over this number is therefore the mean impact interval, in Pluto years; this number times 250 gives the impact interval in Earth years.

The entirety of SP, which covers some 2.5% of the surface of Pluto, has been imaged at the relatively low resolution of 400 m/pixel. No craters are evident in this data set, giving a conservative result that no craters larger than 2 km exist in SP (using 5 pixels conservatively as the detection limit). A small fraction of SP, amounting to around 0.13% of the surface of Pluto, has been imaged at the relatively high resolution of 125 m/pixel. No craters are evident in this data set either, giving a conservative result that no craters larger than ∼625 m exist in this small region of SP. We correct the impact interval above by this surface coverage fraction (1/2.5% and 1/0.13%, respectively).

Recent detailed work on the cratering behavior on Pluto [2] predicts that *D*, the final crater diameter, is proportional to *d*^0.783^, where *d* is the impactor diameter. The 2 km crater detection limit in the low resolution data corresponds roughly to 400 m impactors, while the 625 m crater detection limit in the high resolution imagery corresponds roughly to 90 m impactors.

## Results and Discussion

**Fig 1:**
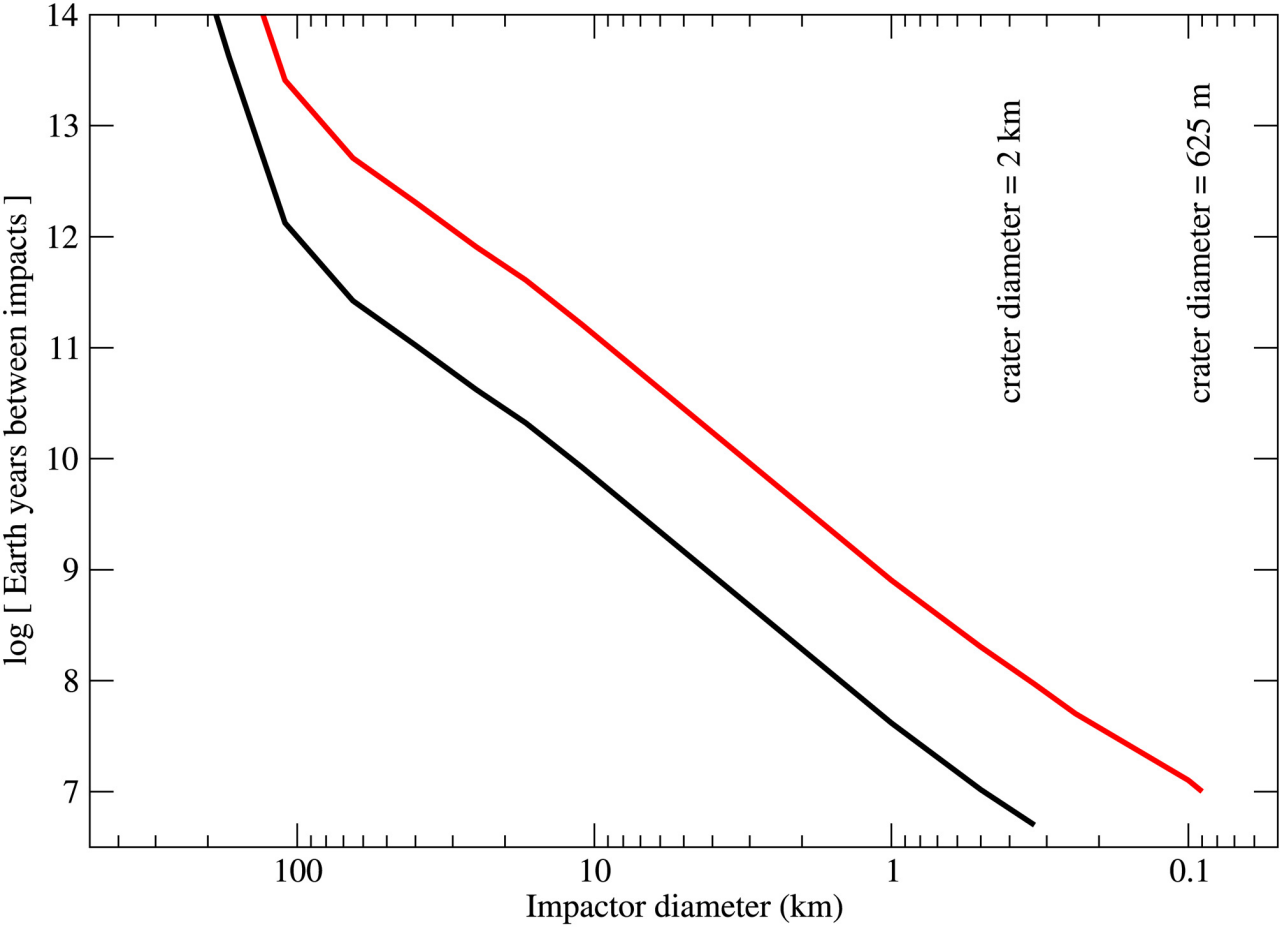
Impact interval onto Sputnik Planum, Pluto, in Earth years as a function of impactor size. The black line shows the constraint from the low resolution imaging (400 m/pixel), which has greater areal coverage; the red line shows the constraint from the small amount of high resolution (125 m/pixel) imaging available at the time of writing. The size distribution of KBOs larger than 10 km is taken from [5]. For sizes smaller than 10 km, estimates and extrapolations from [7] are used. Impactors of size 400 m and 90 m would create craters with the indicated sizes (as derived from scaling laws presented in [2]), which would be at the resolution limit of the low and high resolution imaging, respectively. We conclude that the surface of Sputnik Planum must be younger than around 10 million Earth years. This conclusion is drawn both from the fact that no craters 2 km in diameter have been detected in the low resolution data and no craters 625 meters in diameter have been detected in the smaller amount of high resolution imagery. The two measurements give the same constraint for the maximum surface age of Sputnik Planum.

The imagery requires that no craters caused by 400 m (low resolution) or 90 m (high resolution) impactors exist in SP. Given the known impact interval as a function of size (from above), we can estimate the maximum surface age of SP. The results are shown in Fig 1. The (black, red) lines show the constraints provided by the (low, high) resolution images. In both cases, the conclusion is that the surface age of SP must be less than around 10 million years.

This maximum surface age is surprisingly young and implies that this area of Pluto must be undergoing active resurfacing, presumably through some cryo-geophysical process. There are at least three potential mechanisms by which craters could be erased from SP. The following discussion is largely adapted from [8].

It is possible that craters in SP undergo *viscous relaxation*, in which the surface material flows to relieve any topographic features and horizontal and vertical stresses. The viscous relaxation timescale *τ*_*R*_—the time it takes for the height of a surface feature to diminish by a factor of 1/*e*—is given approximately by 3*η*/*ρgw*, where *η* is the effective viscosity, *ρ* is the density, *g* is the gravitational acceleration, and *w* is the breadth of the depression (in this case, a crater). The density of Pluto’s nitrogen ice is around 1000 kg/m^3^[9] and the gravitational acceleration on Pluto is around 0.66 m/s^2^. To cause a 625 meter crater (the smallest size detectable in the data) to relax over 10^7^ years therefore requires an effective viscosity of the SP surface layer material, which is largely nitrogen ice, of around 4 × 10^19^ Pa-s. Because the relaxation timescale is an upper limit (the surface must be younger than 10^7^ years), the actual effective viscosity must be equal to or less than this value. This is a relatively loose constraint on viscosity; a tighter constraint arises from the next interpretation.

A second possibility is that craters in SP are erased and the surface reset through *convective overturn*. The physics of this anomaly correction is similar to that of viscous relaxation, except that the proximate cause is now a temperature difference from the bottom to the top of the convective cell. This temperature difference causes a density anomaly *Δρ*. The overturn timescale *τ*_*overturn*_ is therefore approximately *η*/*ΔρgL*, where *L* is now the vertical dimension. The difference in density between nitrogen ice at 40 K (Pluto surface temperature) and 60 K (the temperature near the base of the “cryo-lithosphere” just below the nitrogen melting temperature) is around 5% [9], so we use this value for *Δρ*. We assume that the “cell boundaries” seen in the images indicate the horizontal extent of the convection cells—around 30 km—and that the vertical size of a convection cell is around three times smaller than the horizontal extent, or around 10 km. We find a viscosity of equal to or less than around 10^17^ Pa-s. We have not taken into account the stress dependence of the effective viscosity, which would lower our estimate somewhat. Nevertheless, this result is consistent with the viscosity of nitrogen ice at 45 K derived by [10] of around 10^8^ Pa-s, and indicates that convective overturn is a plausible mechanism for removing craters of this size on SP.

A third possible mechanism to erase craters on SP is through *cryovolcanism* that conveys melt from a subsurface reservoir. The assumption here is that at the base of the SP surface layer, which we again take to be on the order of 10 km, there is (perhaps partial) melting of nitrogen ice. This liquid material, which is under pressure, could be extruded to the surface through local cracks (presumably the same cell boundaries described above) and fill in any negative topography before freezing. This mechanism requires that the temperature at the base of the SP surface layer be around 63 K, at which temperature solid nitrogen melts, compared to the surface temperature of 38 K. This implies a temperature difference of around 25 K (the temperature difference across the SP surface layer) over a vertical distance of around 10 km, for a thermal gradient of around 2.5 K/km. The volume of infill material extruded in 10^7^ years must equal the volume of the crater that is erased, which is roughly *πD*^3^/80, where the factor of 10 in the denominator arises from the typical depth/diameter ratio of 1:10. The melt production rate must therefore be around 1 m^3^/year or 10^7^ m^3^ (0.01 km^3^) in ten million years in order to erase a single crater of order 625 meters in diameter.

A last uncertainty in the above constraints is that the size distribution of KBOs at 100 meters is not well known. In our estimate here we have used a reasonable extrapolation from [7]. However, the number of KBOs at that size range could plausibly be a factor of ten greater [11], which makes the surface age of SP younger by that same factor. Alternately, if the number of small KBOs is less than we assumed (for instance, as in [12]), then the surface age increases by the same factor. The estimate used here is an appropriate middle ground.

## Conclusions and Future Work

We have used knowledge of the Kuiper Belt from telescopic observations to constrain the age of Sputnik Planum, Pluto. In the future, additional high-resolution imaging of SP as well as well-characterized crater counting on Pluto’s surface could be used to constrain the small size end of the KBO population. In particular, a better understanding of the crater detection limit in SP will help constrain the number of KBOs smaller than 100 meters. Alternately, very deep and well-characterized surveys for small KBOs might place interesting constraints on the cryo-geophysics of Pluto.
